# Supplementary material for: Structure-guided engineering enables E3 ligase-free and versatile protein ubiquitination via UBE2E1
Source: Nat Commun. 2024 Feb 10;15:1266. doi: 10.1038/s41467-024-45635-y (PMC10858943; doi:10.1038/s41467-024-45635-y)
Supplement: Supplementary file 1 — Supplementary Information file [file 41467_2024_45635_MOESM1_ESM.pdf]

Structure-Guided Engineering Enables E3 Ligase-Free and Versatile Protein Ubiquitination via UBE2E1

Xiangwei Wu[1,2,5], Yunxiang Du[1,5], Lu-Jun Liang[3,5,*], Ruichao Ding[1], Tianyi Zhang[1], Hongyi Cai[1], Xiaolin Tian[4], Man Pan[2,*] & Lei Liu[1,*]

[1] New Cornerstone Science Laboratory, Tsinghua-Peking Joint Center for Life Sciences, MOE Key Laboratory of Bioorganic Phosphorus Chemistry and Chemical Biology, Center for Synthetic and Systems Biology, Department of Chemistry, Tsinghua University, Beijing 100084, China.

[2] Institute of Translational Medicine, School of Chemistry and Chemical Engineering, National Center for Translational Medicine (Shanghai), Shanghai Jiao Tong University, Shanghai, 200240, China.

[3] Center for BioAnalytical Chemistry, Hefei National Laboratory of Physical Science at Microscale, University of Science and Technology of China, Hefei 230026, China

[4] MOE Key Laboratory of Bioinformatics, School of Life Sciences, Tsinghua University, 100084 Beijing, China

[5] These authors contributed equally to this work: X. Wu, Y. Du, L. Liang.

[*]To whom correspondence should be addressed: Lei Liu (lliu@mail.tsinghua.edu.cn); Man Pan (panman@sjtu.edu.cn), Lu-Jun Liang (lujun@ustc.edu.cn).

Supplementary Figure 1-14

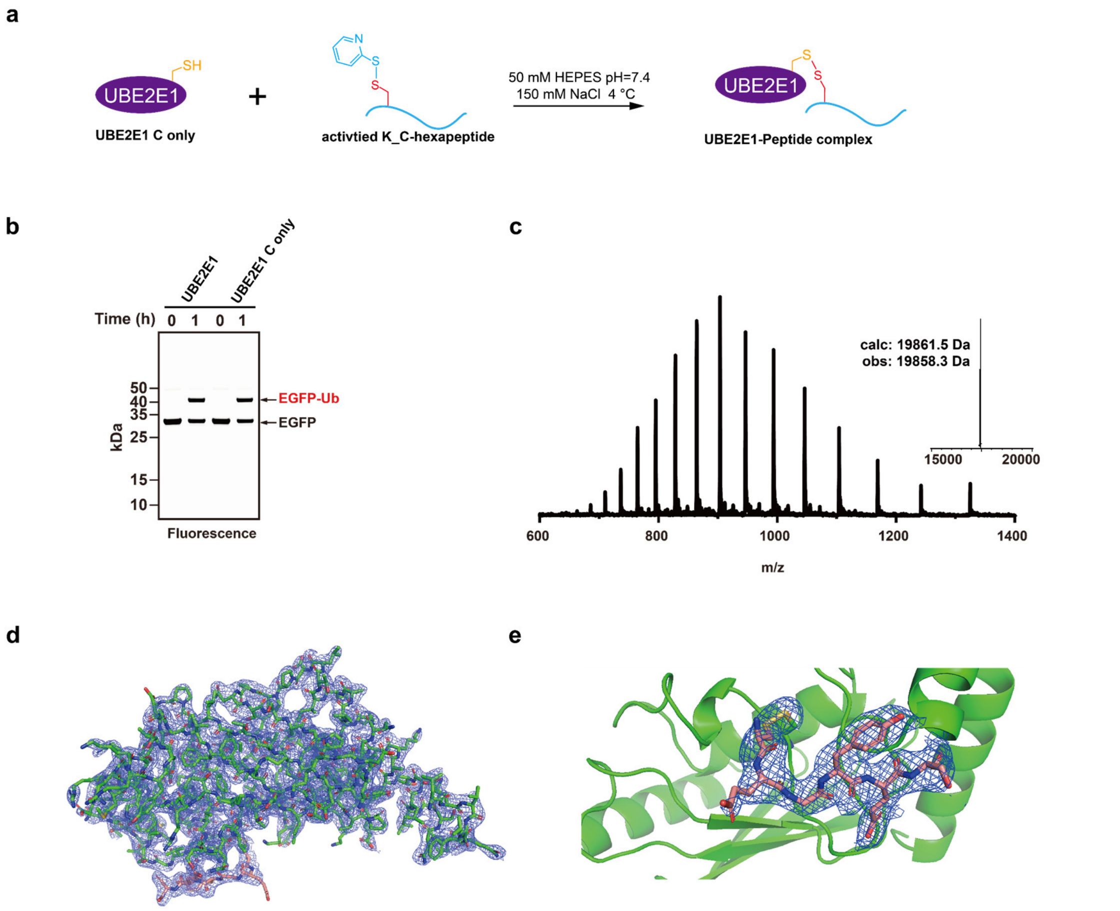

Supplementary Figure 1. Crystal structure of UBE2E1/SEDTB1-derived peptide complex.

a, The synthetic route to obtain the UBE2E1-peptide complex, in which the lysine on the hexapeptide was mutated to cysteine to enable a disulfide bond coupling with UBE2E1 mutant containing only one cysteine in the active center (UBE2E1 C only). The preparation process of activtied K_C-hexapeptide was shown in Supplementary Figure 11. **b**, The UBE2E1 mutant (UBE2E1 C only) possesses the activity to catalyze ubiquitination reaction. These E3-free ubiquitination assays were performed with 1 µM Uba1, 5 µM UBE2E1 or UBE2E1 C only, 80 µM Ub, and 8µM peptide (KEGYES)-fused EGFP. Gel images are representative of independent biological replicates (n = 2). **c**, Representative ESI-MS traces of UBE2E1-peptide covalent complex. The observed (obs.) and calculated (calc.) molecular weights were marked.

d, The electron density 2Fo–Fc of the crystal complex is shown in blue mesh contoured

at 1.0 σ. UBE2E1 is shown as sticks and colored in green. The peptide is shown as sticks and colored in salmon. **e**, The electron density 2Fo–Fc of the peptide ligand is shown in blue mesh contoured at 0.8 σ. Source data are provided as a Source Data file.

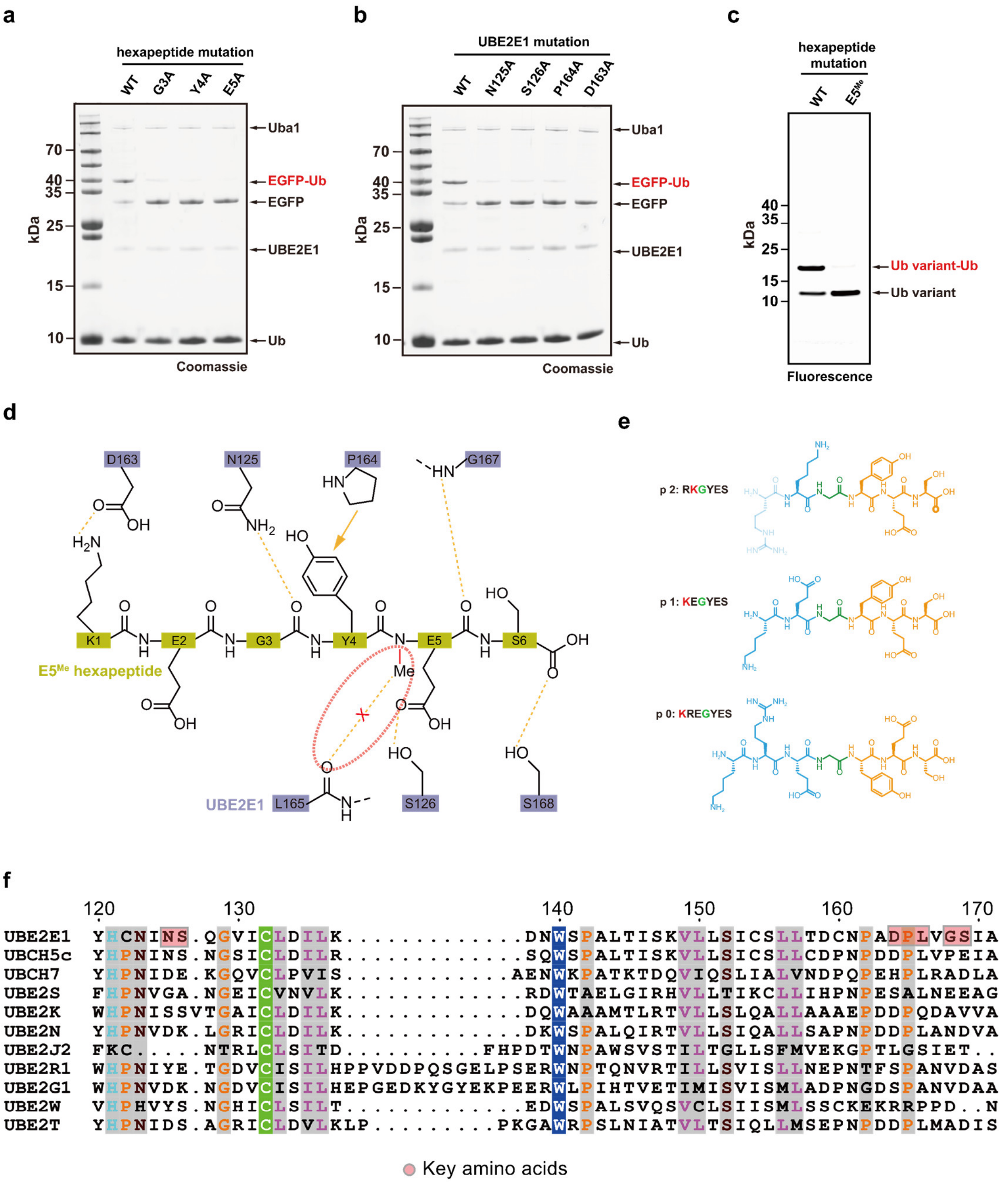

Supplementary Figure 2. Key residues governing hexapeptide recognition by UBE2E1.

a, In vitro E3-free Ubiquitination assay using hexapeptide mutants fused EGFP as substrates. **b**, In vitro E3-free Ubiquitination assay using UBE2E1 mutants with EGFP* as substrates. **c**, The chemically synthesized substrate (a ubiquitin variant with E5^{Me} hexapeptide) failed to be ubiquitinated by UBE2E1. The preparation process of those substrates was shown in Supplementary Figure 12. **d**, Schematic representation of methylation on the α-amino group of E5 (E5^{Me}) perturbing its interaction with L165 of UBE2E1. **e**, The proper distance between the receptor lysine and glycine at the corner

of “L-shaped” hexapeptide is necessary for ubiquitination. **f**, The complete interaction network required for the recognition of hexapeptides is specific to UBE2E1. E2s, with different preferences for ubiquitin chain linkages, were selected to compare with UBE2E1. Gel images shown in (a)-(c) are representative of independent biological replicates (n = 2). Source data are provided as a Source Data file.

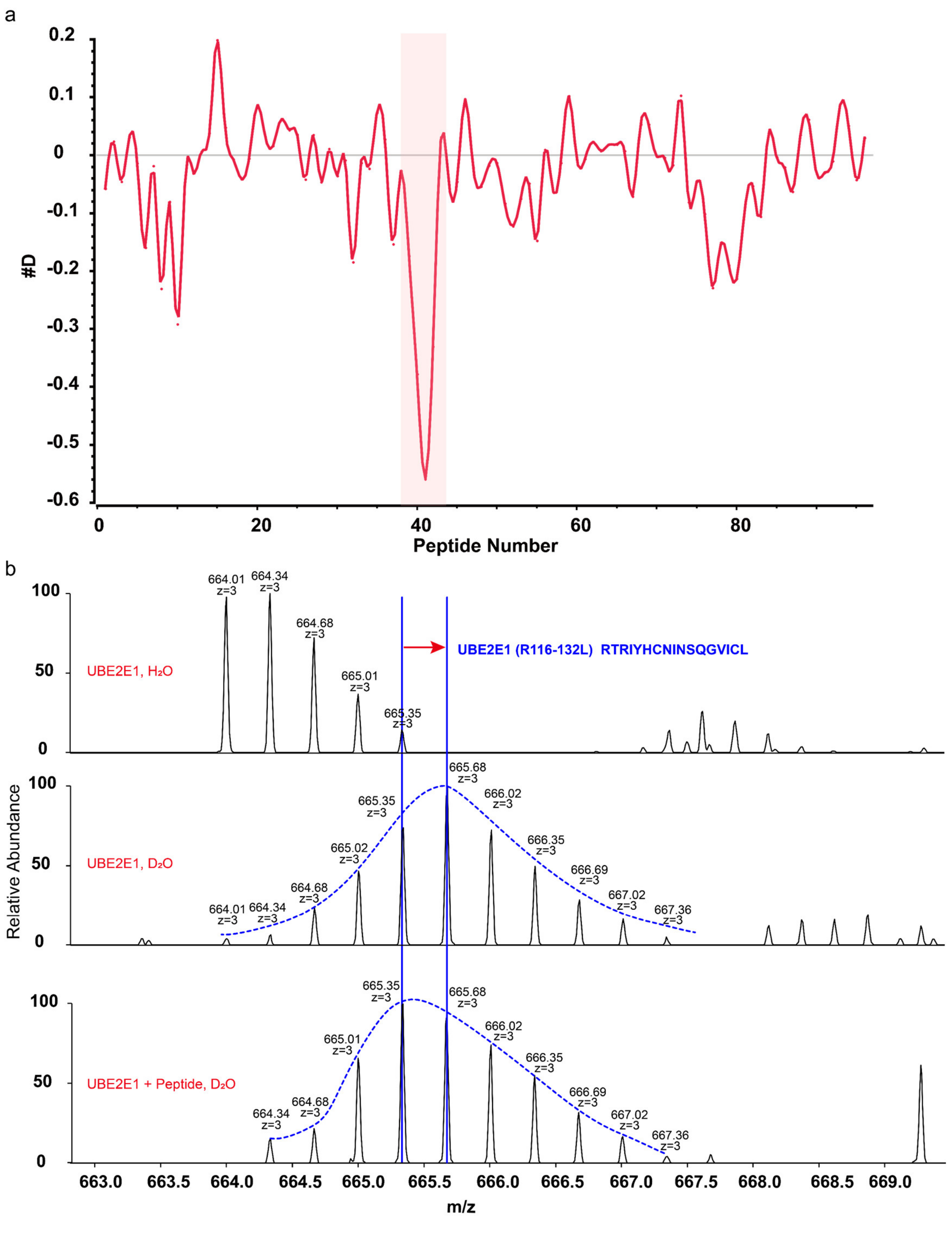

Supplementary Figure 3. HDX-MS assay

a, HDX-MS difference map of UBE2E1 in the presence and absence of the peptide (KEGYES) at deuterium exchange time of 90 seconds. Changes in deuterium incorporation corresponding to the UBE2E1 (116-132) fragment are highlighted by red shade. **b**, HDX-MS results of UBE2E1 (116-132) in the presence and absence of

the peptide (KEGYES). The centroid mass changes between the two states as indicated in the red arrow.

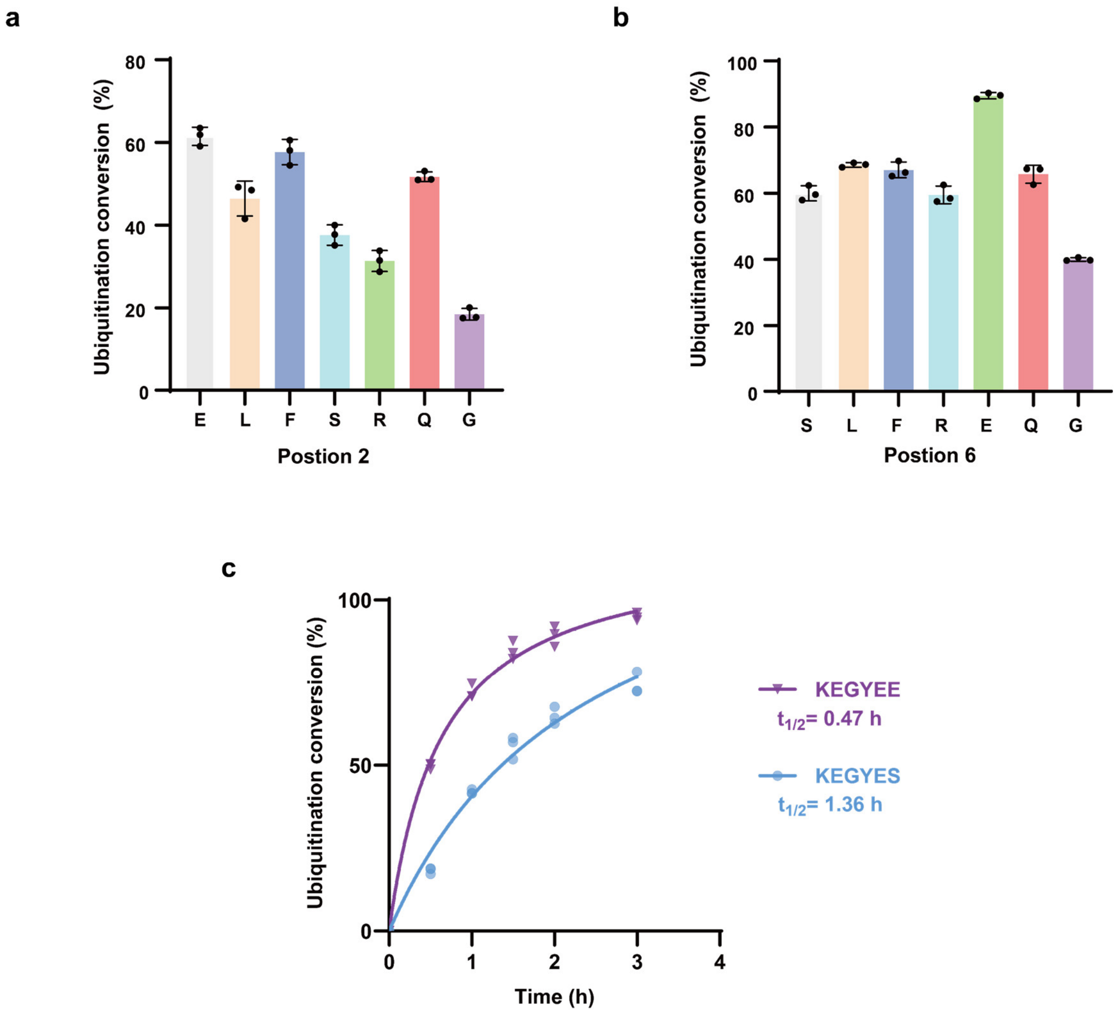

Supplementary Figure 4. SUE1 tag with stronger ubiquitination activity.

a, In vitro E3-free Ubiquitination assay using EGFP fused hexapeptide bearing mutation at position 2 (KXGYES) as substrates. The ubiquitination conversion was determined by the gel density and data represent the mean ± SD of three independent experiments. **b**, In vitro E3-free Ubiquitination assay using EGFP fused hexapeptide bearing mutation at position 6 (KEGYEX) as substrates. The ubiquitination conversion was determined by the gel density and data represent the mean ± SD of three independent experiments. **c**, The ubiquitination conversion over time of SUE1 tag fused EGFP. The apparent ubiquitination reaction half-life ($t_{1/2}$) is marked. These data were from independent biological replicates (n= 3). Source data are provided as a Source Data file.

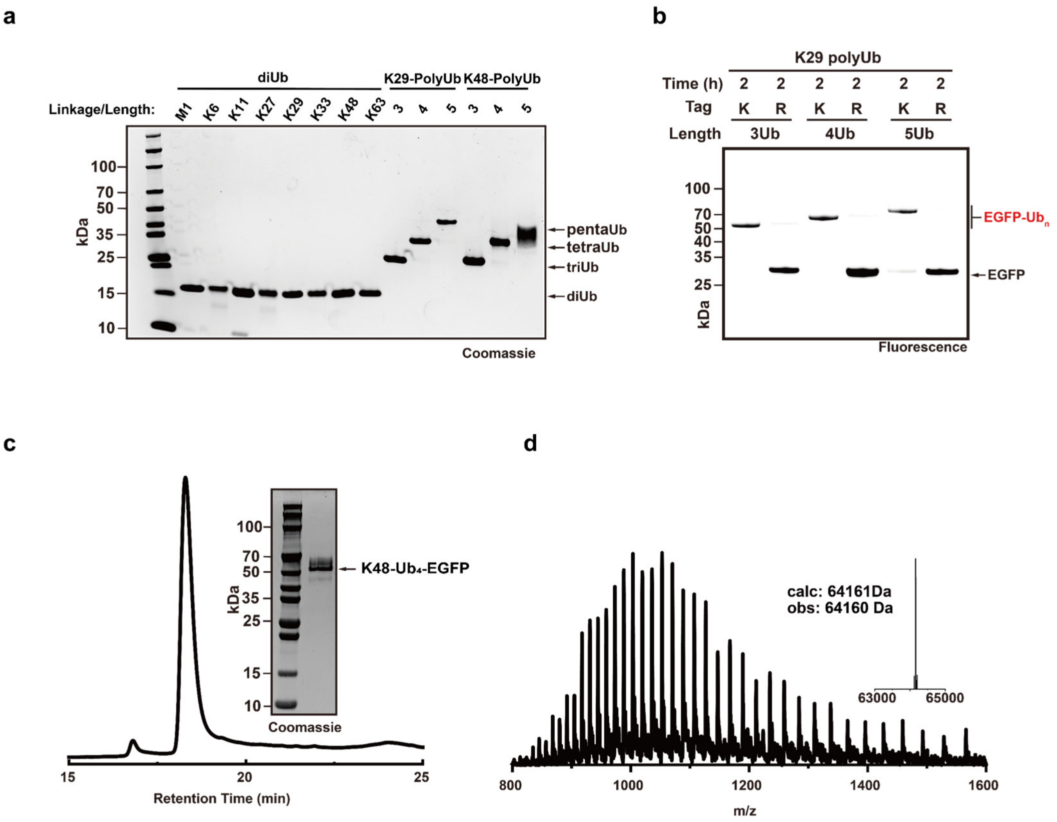

Supplementary Figure 5. Access to ubiquitinated substrates using the SUE1 strategy.

a, SDS–PAGE analysis of ubiquitin chains used in the SUE1 strategy. **b**, The SUE1 strategy enables access to K29 linked-polyUb-modified substrates with different lengths (n= 3/4/5). Gel images are representative of independent biological replicates (n = 2). **c**, HPLC and SDS–PAGE analysis of purified K48 linked tetra-ubiquitin chain-modified EGFP (K48-Ub_4-EGFP). **d**, Representative MS traces of K48-Ub_4-EGFP. The observed (obs.) and calculated (calc.) molecular weights were marked. Source data are provided as a Source Data file.

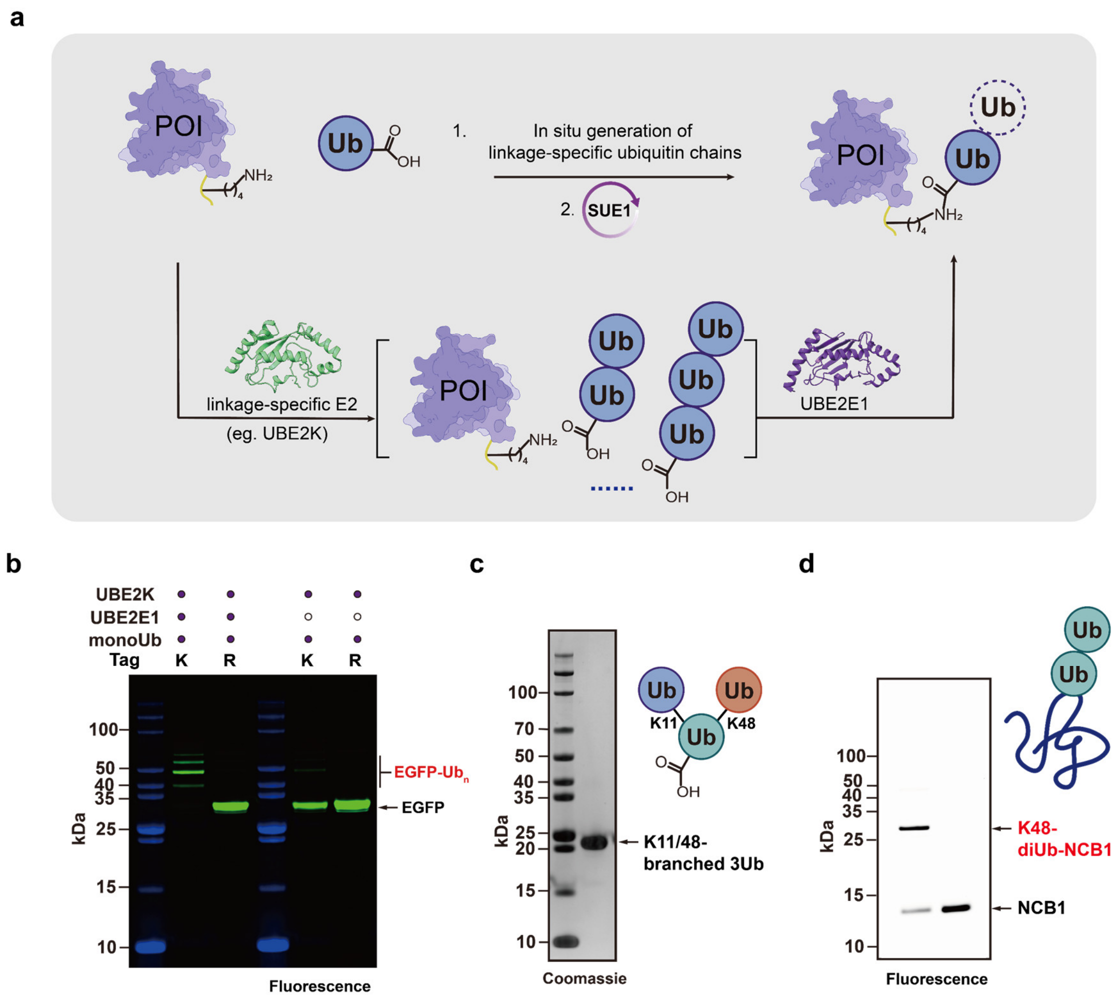

Supplementary Figure 6. SUE1 strategy utilizing pure or mixed ubiquitin chains and branched ubiquitin chains.

a, Schematic representation of SUE1 enabling the transfer of ubiquitin chain in situ generated by linkage-specific E2 enzymes to the substrate. **b**, SDS–PAGE analysis of in situ generated ubiquitin chains transferred to substrates by SUE1. **c**, SDS–PAGE analysis of K11/48-branched triUb used in the SUE1 reaction. **d**, The SUE1 strategy to obtain K48 linked-diUb-modified NCB1. Mutation of the ubiquitination modification site K64 to R64 as a negative control. Gel images shown in (b) and (d) are representative of independent biological replicates (n = 2). Source data are provided as a Source Data file.

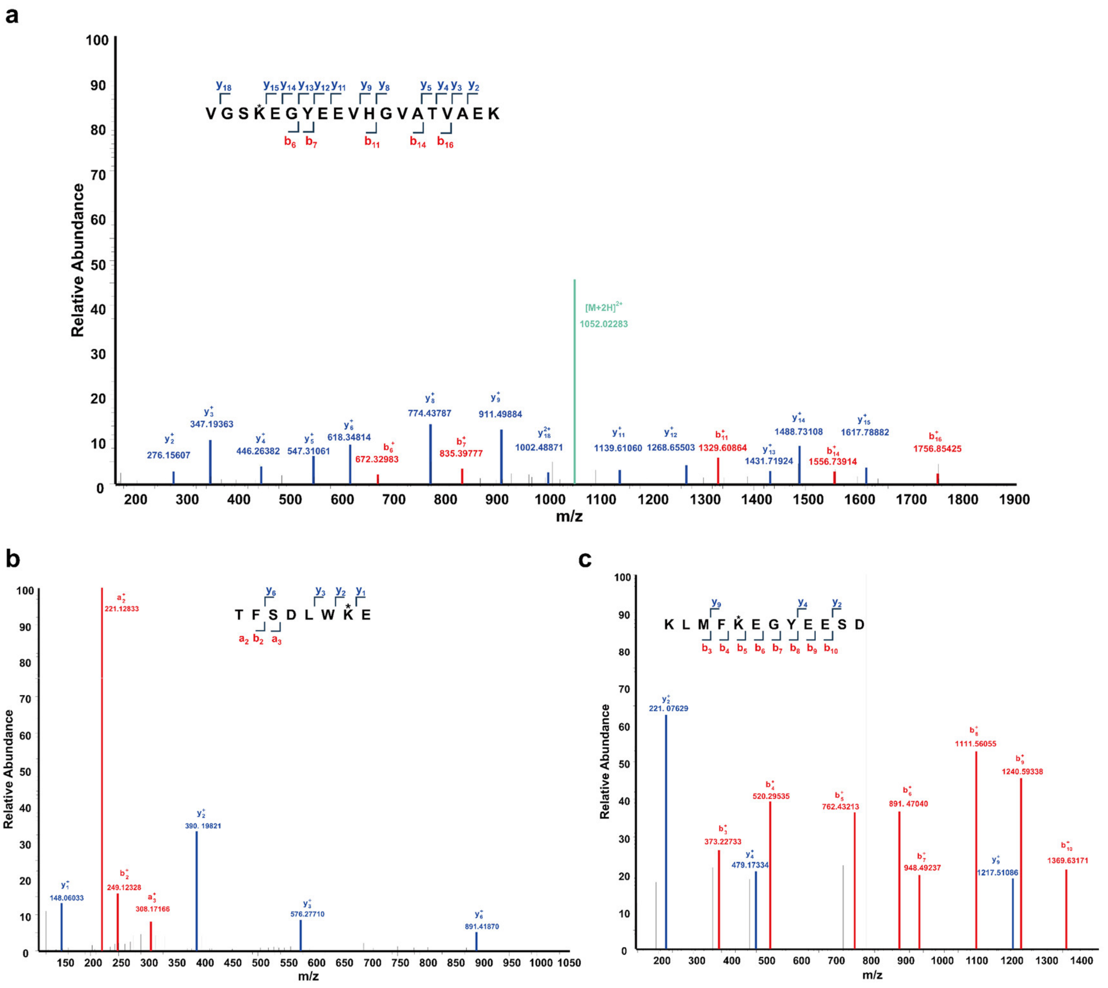

Supplementary Figure 7. LC–MS/MS analysis of the ubiquitination sites of the protein.

a, MS/MS spectrum of the detected peptide VGSKEGYEEVHGVATVAEK from α-syn-Ub sample with a glycine-glycine (GG) branch on the first lysine. **b**, MS/MS spectrum of the detected peptide TFSDLWKE from p53-Ub sample with a glycine-glycine (GG) branch on the lysine. **c**, MS/MS spectrum of the detected peptide KLMFKEGYEESD from p53-diUb sample with a glycine-glycine (GG) branch on the second lysine.

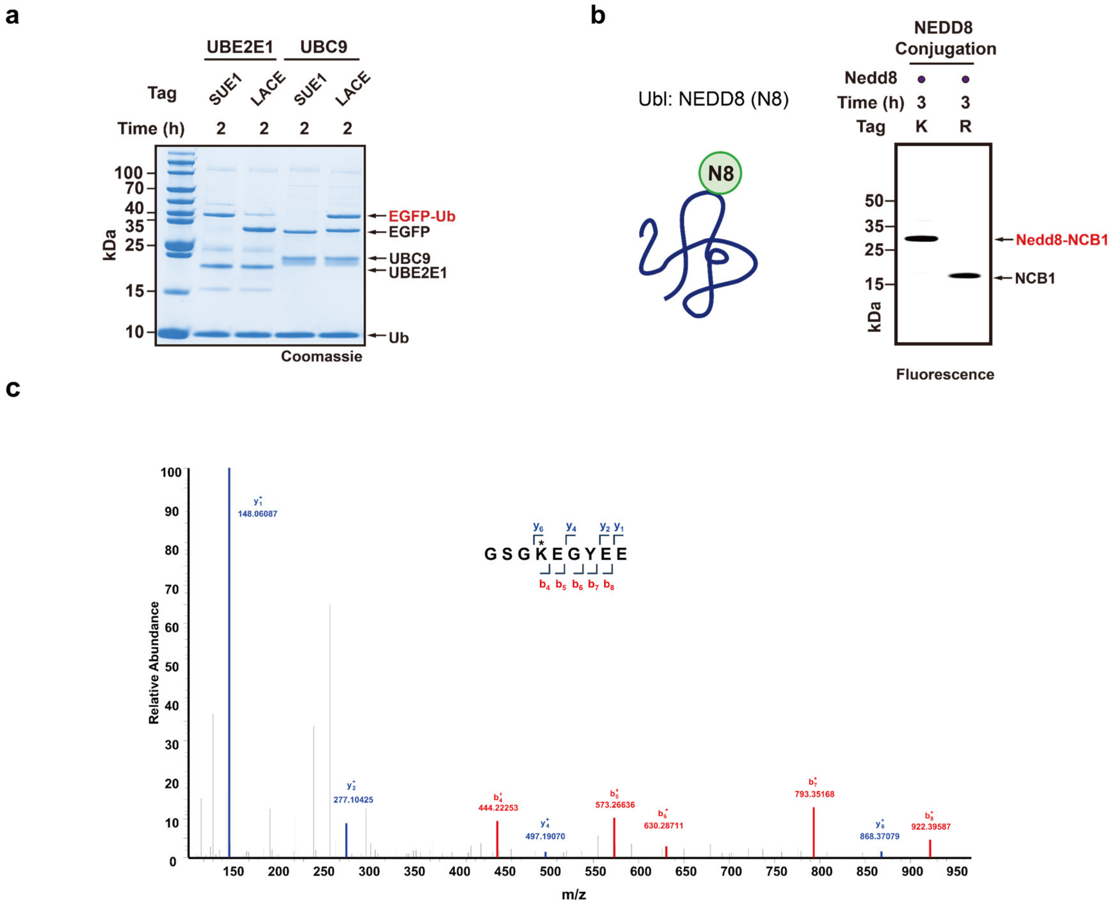

Supplementary Figure 8. Orthogonality assay and acquisition of precise NEED8-modified proteins.

a, Orthogonality assay using mutually unmatched enzyme-tag pairs. **b**, SDS–PAGE analysis of NEDD8-modified NCB1 generated by the SUE1 strategy. **c**, MS/MS spectrum of the detected peptide GSGKEGYEE from the EGFP-N8 sample with a glycine-glycine (GG) branch on the second lysine. Gel images shown in (a) and (b) are representative of independent biological replicates (n = 2). Source data are provided as a Source Data file.

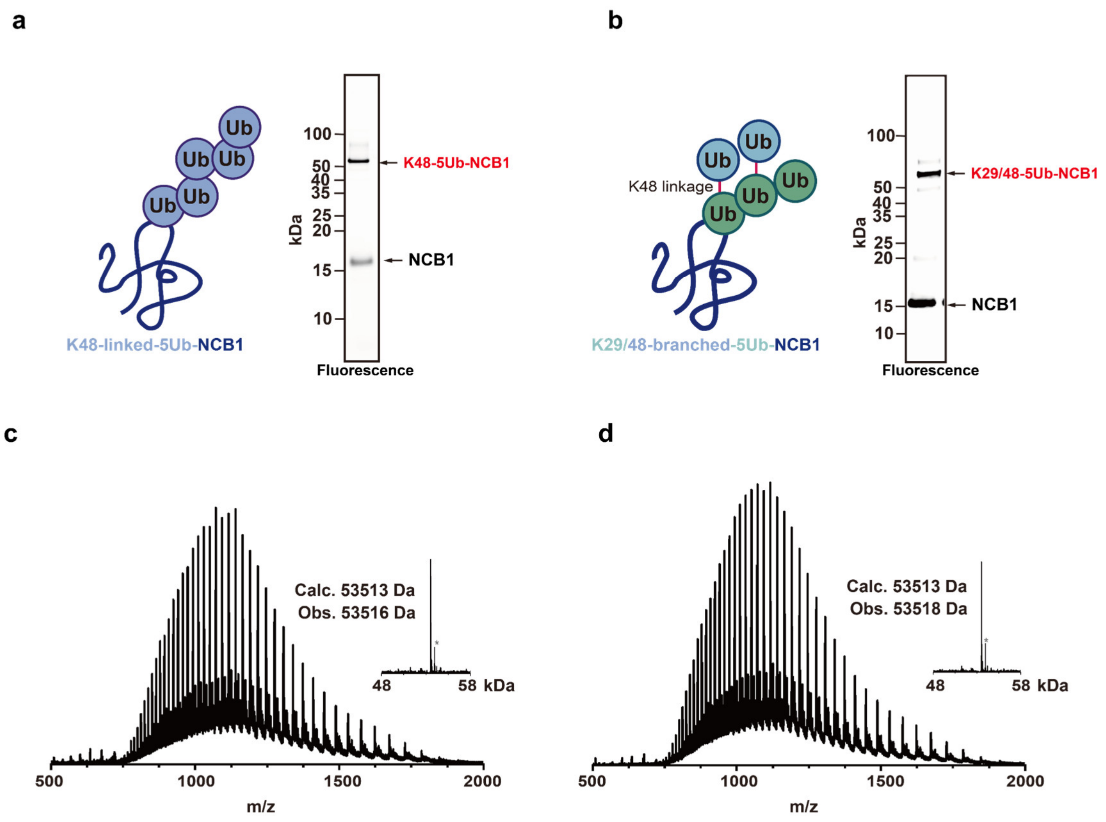

Supplementary Figure 9. Ubiquitinated NCB1 for in vitro degradation assay.

a, SDS–PAGE analysis of K48-linked pentaUb-modified NCB1 (K48-Ub_5-NCB1) obtained by the SUE1 strategy. **b**, SDS–PAGE analysis of K29/48-branched pentaUb modified NCB1 (K29/48-Ub_5-NCB1) obtained by the SUE1 strategy. **c**, Representative MS traces of fluorescently labeled K48-Ub_5-NCB1 and partial product (*) was labeled with two fluorescent groups (+445 Da). **d**, Representative MS traces of fluorescent labeled K29/48-Ub_5-NCB1 and partially product (*) was labeled with two fluorescently groups (+445 Da). Gel images shown in (a) and (b) are representative of independent biological replicates (n = 2). Source data are provided as a Source Data file.

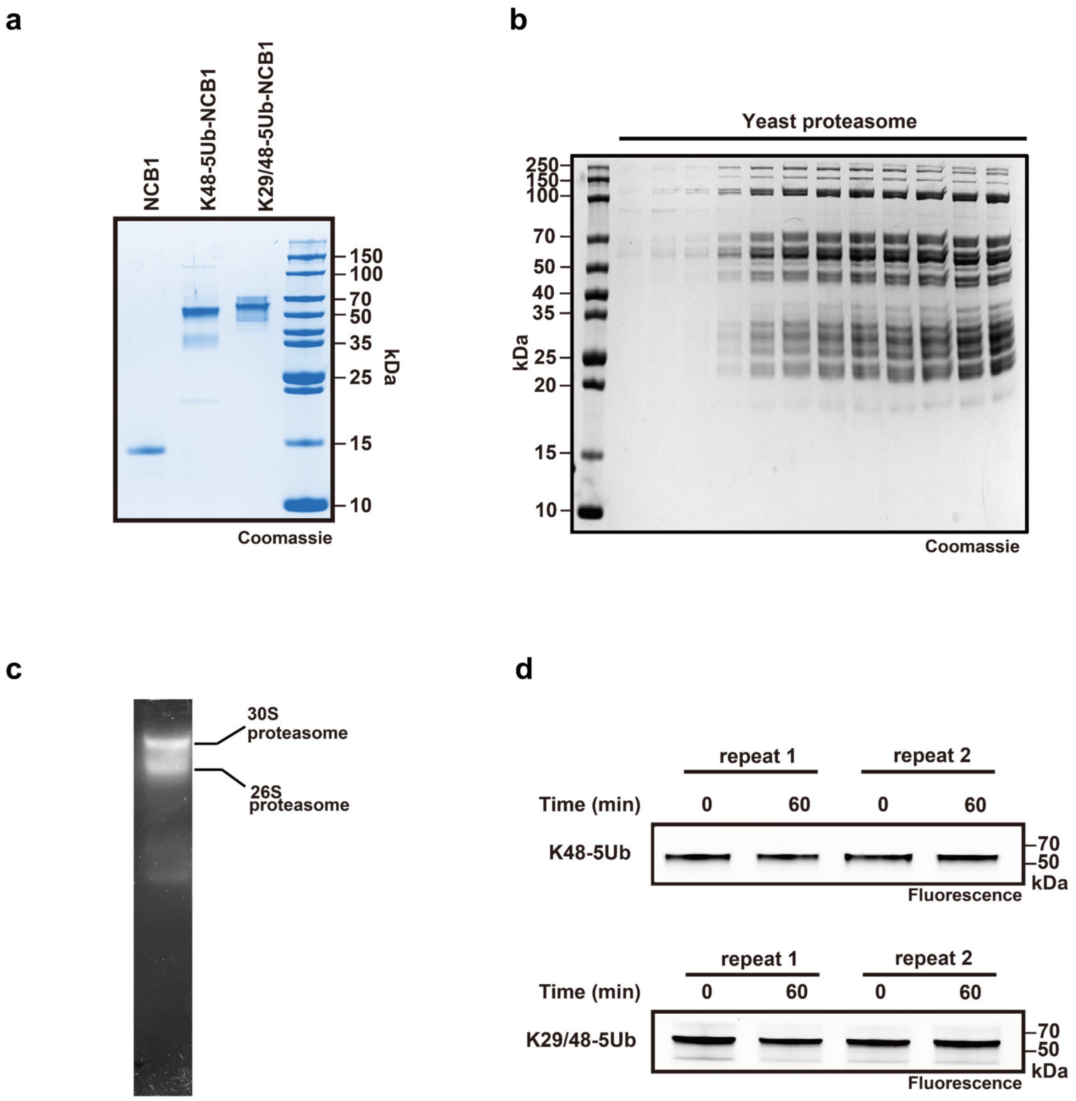

Supplementary Figure 10. In vitro degradation assay.

a, SDS–PAGE analysis of purified ubiquitinated modified NCB1. **b**, SDS–PAGE analysis of the yeast 26S proteasome. **c**, Native gel analysis of peptidase activity using Suc-LLVY-AMC. The 30S proteasome (doubly capped) and 26S proteasome (single capped) are indicated. Gel images shown in (c) are representative of independent biological replicates (n = 2). **d**, Ubiquitinated NCB1 is maintained stably in the absence of proteasome. Two independent biological replicates were conducted. Source data are provided as a Source Data file.

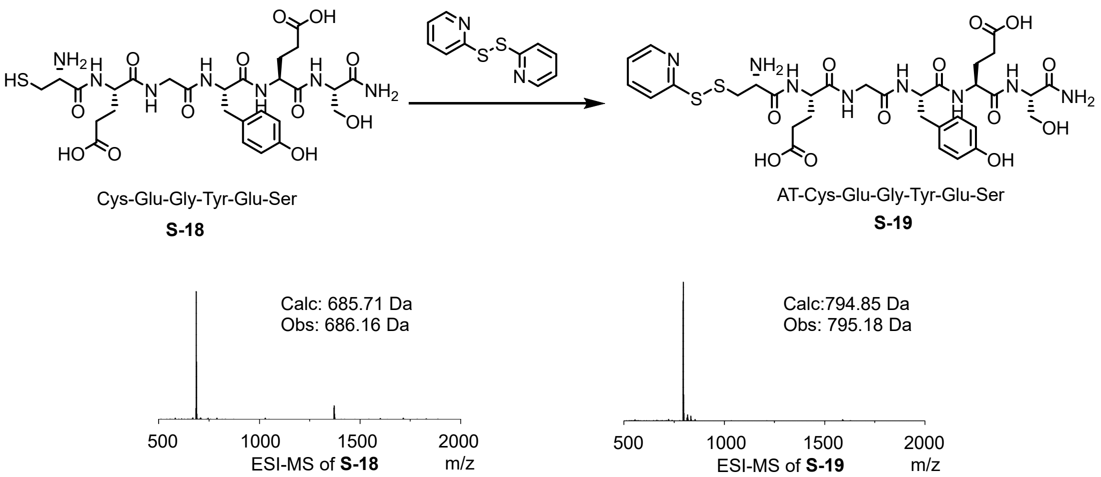

Supplementary Figure 11. Chemical synthesis of disulfide bond activated hexapeptide.

Synthetic route of AT-CEGYES-NH_2 and ESI-MS of starting material and final product.

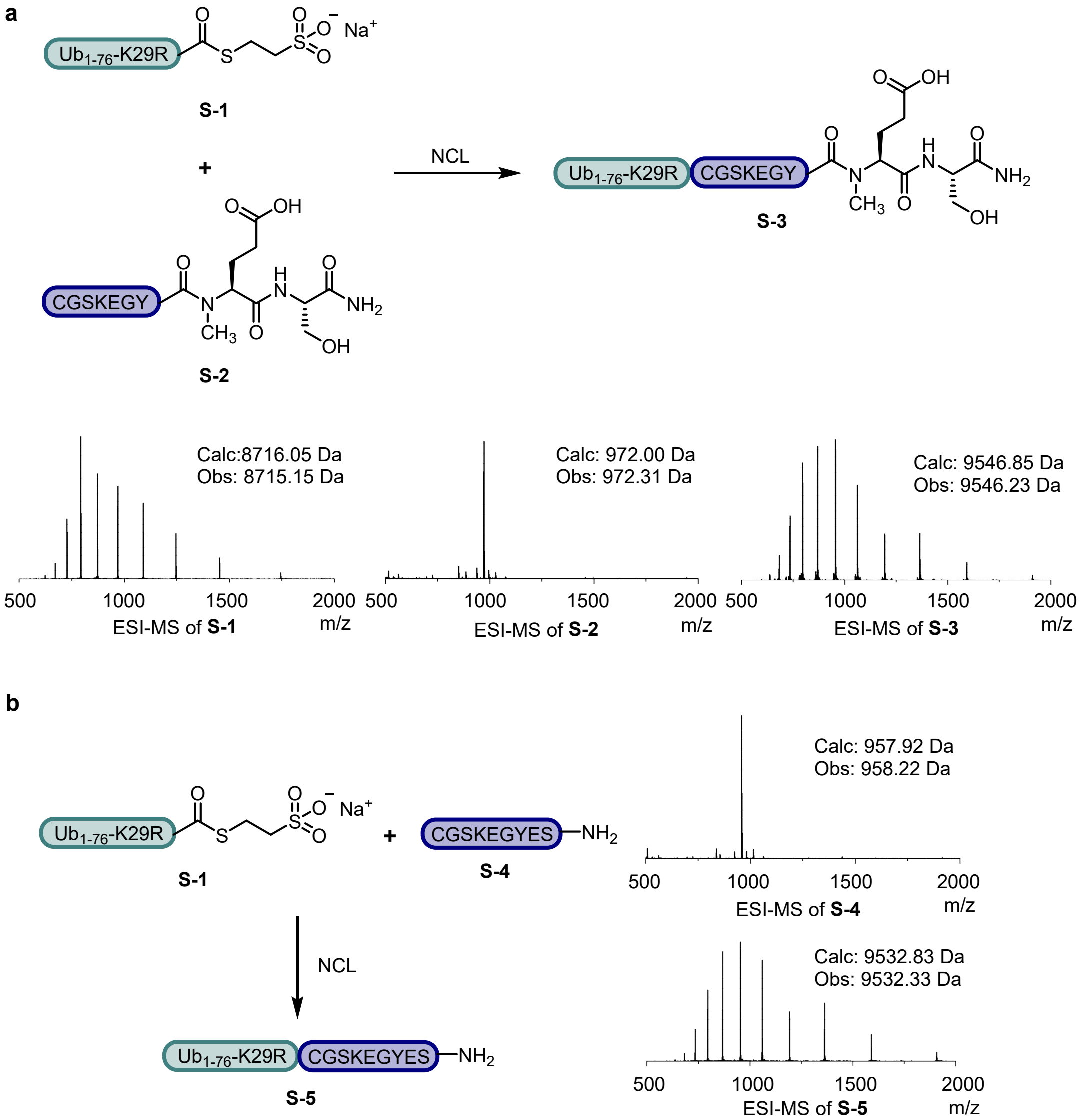

Supplementary Figure 12. Chemical synthesis of model substrates for investigating interaction between UbE2E1 and KEGYES sequence.

a, Synthetic route of $Ub_{1\text{-}76}$-K29R-CGSKEGYE(α-N(Me))S-NH_2 and ESI-MS of intermediates and final product. **b**, Synthetic route of $Ub_{1\text{-}76}$-K29R-CGSKEGYES-NH_2 and ESI-MS of intermediates and final product.

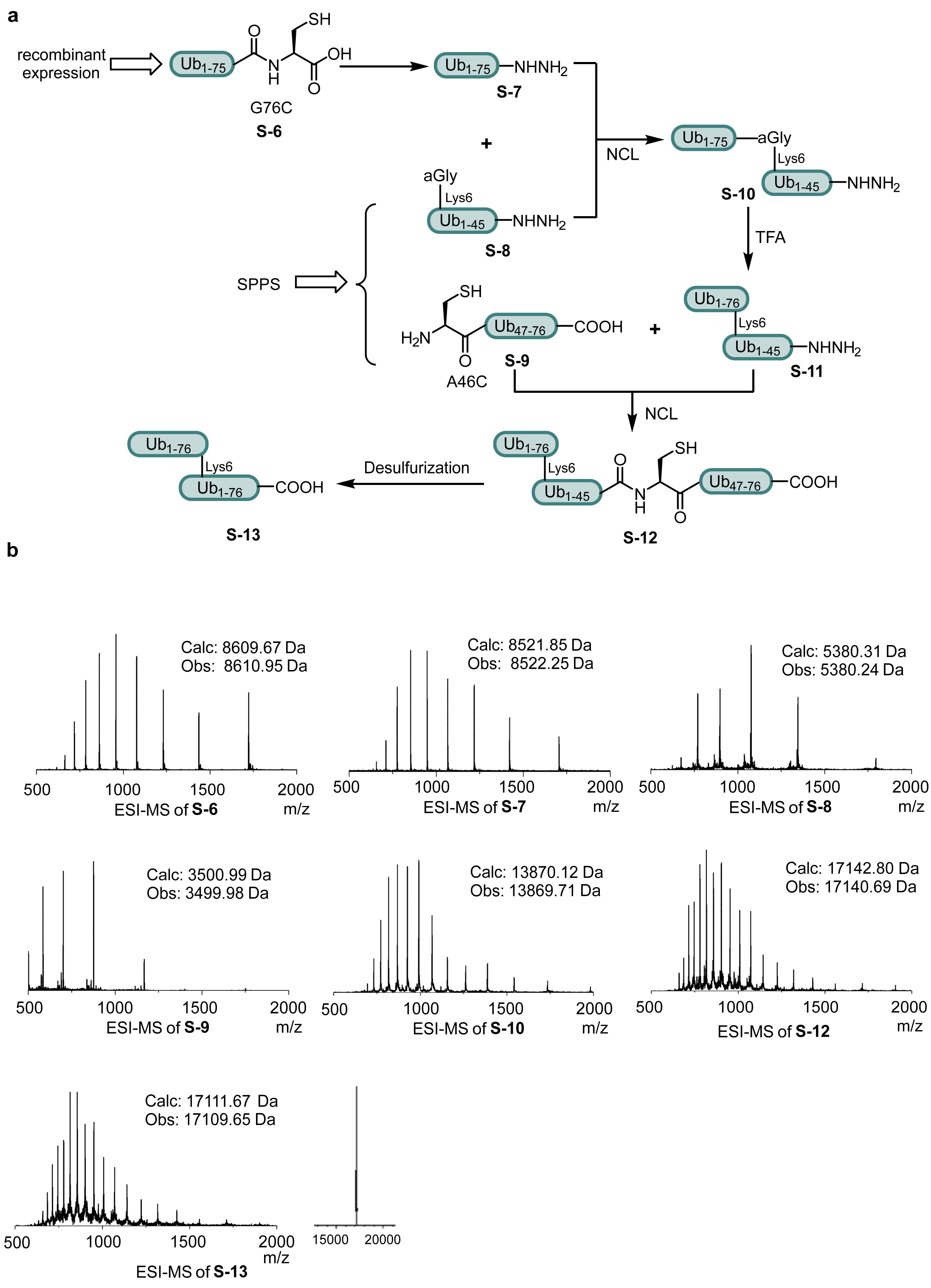

Supplementary Figure 13. Chemical synthesis of K6 diUb.

a, General synthetic route of K6 diUb. **b**, ESI-MS of intermediates and final product.

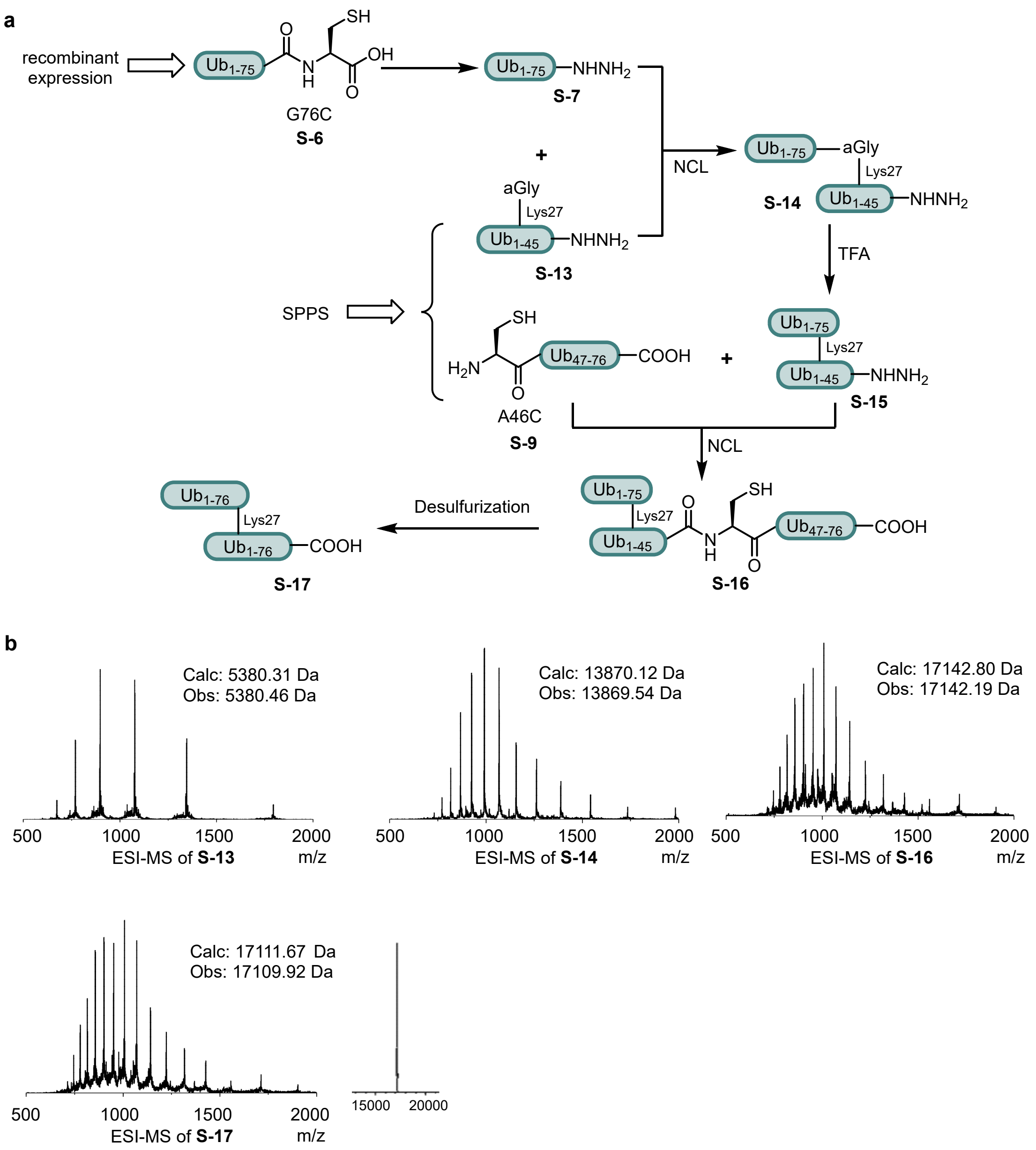

Supplementary Figure 14. Chemical synthesis of K27 diUb.

a, General synthetic route of K27 diUb. **b**, ESI-MS of intermediates and final product.

Supplementary Table 1-5

Supplementary Table 1. Crystallographic data collection and refinement statistics.

	UBE2E1-SETDB1 derived peptide[†] (PDB 8IYA)
**Data collection**	
Space group	C121
Cell dimensions	
a, *b*, *c* (Å)	102.75, 50.08, 109.25
α, β, γ (°)	90.0, 91.75, 90.0
Resolution (Å)	45.01 - 2.43 (2.57 - 2.43) [a]
No. of unique reflections	21 079
R_{merge}	0.131 (0.633) [a]
R_{pim}	0.067 (0.311) [a]
R_{meas}	0.148
I/(*I*)	7.0 (2.24) [a]
$CC_{1/2}$	0.989 (0.877) [a]
Completeness (%)	99.7 (99.9) [a]
Redundancy	4.6 (4.8) [a]
**Refinement**	
Resolution (Å)	2.43
No. reflections	21 070
R_{work} / R_{free} (%)	20.4 / 24.6
No. of non-hydrogen atoms	3913
Protein	3862
Ligand/ion	15
Water	36
B factors (Å^2)	53.0 (Average)
Protein[b]	52
Ligand/ion[b]	30
Solvent	42.8
R.m.s. deviations	
Bond lengths (Å)	0.009
Bond angles (°)	1.136
Clashscore	7.2

[a] Values in parentheses are for highest-resolution shell.

[b]The median values of the occupancy-weighted average B-factor per residue.

[†]The data are collected from one crystal.

Supplementary Table 2. Kinetic parameters of UBE2E1-mediated Ubiquitination for original hexapeptide and the S6E peptide-fused EGFP.

Sequence	k_{cat} (min^{-1})	K_m (μM)	k_{cat}/K_m (M^{-1} s^{-1})
KEGYEE	1.13	491.88	38.29
KEGYES	0.45	382.70	19.60

Supplementary Table 3. Materials and reagents for protein chemical synthesis.

materials and reagents	Company
Rink Amide AM resin (0.37mmol/g)	Tianjin Nankai Hecheng Science & Technology Co., Ltd (TianJin, China)
2-Chlorotrityl resin (0.65mmol/g)	Tianjin Nankai Hecheng Science & Technology Co., Ltd (TianJin, China)
ChemMatrix® Resin	Sigma-Aldrich
Fmoc/Boc-amino acids block	GL Biochem., Ltd (Shanghai, China)
N, N'-diisopropylcarbodiimide (DIC)	Adamas-beta (Shanghai, China)
Ethyl cyanoglyoxylate-2-oxime (oxyma)	Adamas-beta (Shanghai, China)
Guanidine hydrochloride (Gn-HCl)	Adamas-beta (Shanghai, China)
Sodium nitrite ($NaNO_2$)	Adamas-beta (Shanghai, China)
4-Mercaptobenzoic acid (MPAA)	Bide Pharmatech Ltd.
Tris(2-carboxyethyl)phosphine hydrochloride (TCEP)	Bide Pharmatech Ltd.
2,2'-Azobis[2-(2-imidazolin-2-yl)propane] dihydrochloride (VA-044)	Bide Pharmatech Ltd.
Phenylsilane	Shanghai Titan Scientific Co.,Ltd
di(tert-butyl) carbonate	Shanghai Titan Scientific Co.,Ltd
Imidazole	Shanghai Titan Scientific Co.,Ltd
2-[4-(2-Hydroxyethyl)-1-piperazinyl] ethanesulfonic Acid (HEPES)	Shanghai Titan Scientific Co.,Ltd
Acetonitrile (HPLC grade)	J. T. Baker (Phillipsburg, NJ, USA)
N, N-Dimethylformamide (DMF)	J&K Scientific (Beijing, China)
trifluoroacetic acid (TFA) (HPLC grade)	J&K Scientific (Beijing, China)
Fluorescein isothiocyanate isomer I (FITC)	J&K Scientific (Beijing, China)
triisopropylsilane (TIPS)	J&K Scientific (Beijing, China)
$NaH_2PO_4 \cdot 12H_2O$	J&K Scientific (Beijing, China)
$Na_2HPO_4 \cdot 12H_2O$	J&K Scientific (Beijing, China)
Dithiothreitol (DTT)	J&K Scientific (Beijing, China)
Palladium Chloride ($PdCl_2$)	J&K Scientific (Beijing, China)
1,2-di(pyridin-2-yl)disulfane	J&K Scientific (Beijing, China)
Sodium diethyldithiocarbamate trihydrate	J&K Scientific (Beijing, China)
Piperidine	Sinopharm Chemical Reagent Co., Ltd
Diethyl ether (Et_2O)	Sinopharm Chemical Reagent Co., Ltd
Ethanedithiol (EDT)	TCI (Shanghai, China) Development Co., Ltd.

Supplementary Table 4. Protein sequences.

Protein name	Amino acid sequence
UBE2E1	MHHHHHHHHHLEVLFQGPNSKLLSTSAKRIQKELADITLDPPPNCSA<br>GPKGDNIYEWRSTILGPPGSVYEGGVFFLDITFTPEYPFKPPKVTFR<br>TRIYHCNINSQGVICLDILRDNWSPALTISKVLLSICSLLTDCNPADPL<br>VGSIATQYMTNRAEHDRMARQWTKRYAT
UBE2E1 C131A	MHHHHHHHHHLEVLFQGPNSKLLSTSAKRIQKELADITLDPPPNCSA<br>GPKGDNIYEWRSTILGPPGSVYEGGVFFLDITFTPEYPFKPPKVTFR<br>TRIYHCNINSQGVICLDILKDNWSPALTISKVLLSICSLLTDCNPADPL<br>VGSIATQYMTNRAEHDRMARQWTKRYAT
UBE2E1 C only	MHHHHHHHHHLEVLFQGPNSKLLSTSAKRIQKELADITLDPPPNSSA<br>GPKGDNIYEWRSTILGPPGSVYEGGVFFLDITFTPEYPFKPPKVTFR<br>TRIYHPNINSQGVICLDILKDNWSPALTISKVLLSISSLLTDPNPADPL<br>VGSIATQYMTNRAEHDRMARQWTKRYAT
UBE2E1 N125A	MHHHHHHHHHLEVLFQGPNSKLLSTSAKRIQKELADITLDPPPNCSA<br>GPKGDNIYEWRSTILGPPGSVYEGGVFFLDITFTPEYPFKPPKVTFR<br>TRIYHCNIASQGVICLDILRDNWSPALTISKVLLSICSLLTDCNPADPL<br>VGSIATQYMTNRAEHDRMARQWTKRYAT
UBE2E1 S126A	MHHHHHHHHHLEVLFQGPNSKLLSTSAKRIQKELADITLDPPPNCSA<br>GPKGDNIYEWRSTILGPPGSVYEGGVFFLDITFTPEYPFKPPKVTFR<br>TRIYHCNINAQGVICLDILRDNWSPALTISKVLLSICSLLTDCNPADP<br>LVGSIATQYMTNRAEHDRMARQWTKRYAT
UBE2E1 P164A	MHHHHHHHHHLEVLFQGPNSKLLSTSAKRIQKELADITLDPPPNCSA<br>GPKGDNIYEWRSTILGPPGSVYEGGVFFLDITFTPEYPFKPPKVTFR<br>TRIYHCNINSQGVICLDILRDNWSPALTISKVLLSICSLLTDCNPADA<br>LVGSIATQYMTNRAEHDRMARQWTKRYAT
UBE2E1 D163A	MHHHHHHHHHLEVLFQGPNSKLLSTSAKRIQKELADITLDPPPNCSA<br>GPKGDNIYEWRSTILGPPGSVYEGGVFFLDITFTPEYPFKPPKVTFR<br>TRIYHCNINSQGVICLDILRDNWSPALTISKVLLSICSLLTDCNPAAPL<br>VGSIATQYMTNRAEHDRMARQWTKRYAT
EGFP[*](EGFP-KEGYES)	MHHHHHHHHHLEVLFQGPMVSKGEELFTGVVPILVELDGDVNGHK<br>FSVSGEGEGDATYGKLTLKFICTTGKLPVPWPTLVTTLTYGVQCFSR<br>YPDHMKQHDFFKSAMPEGYVQERTIFFKDDGNYKTRAEVKFEGD<br>TLVNRIELKGIDFKEDGNILGHKLEYNYNSHNVYIMADKQKNGIKV<br>NFKIRHNIEDGSVQLADHYQQNTPIGDGPVLLPDNHYLSTQSALSK<br>DPNEKRDHMVLLEFVTAAGITLGMDELYKGSG**KEGYES**
EGFP[**](EGFP-REGYES)	MHHHHHHHHHLEVLFQGPMVSKGEELFTGVVPILVELDGDVNGHK<br>FSVSGEGEGDATYGKLTLKFICTTGKLPVPWPTLVTTLTYGVQCFSR<br>YPDHMKQHDFFKSAMPEGYVQERTIFFKDDGNYKTRAEVKFEGD<br>TLVNRIELKGIDFKEDGNILGHKLEYNYNSHNVYIMADKQKNGIKV<br>NFKIRHNIEDGSVQLADHYQQNTPIGDGPVLLPDNHYLSTQSALSK<br>DPNEKRDHMVLLEFVTAAGITLGMDELYKGSG**REGYES**
EGFP-G3A	MHHHHHHHHHLEVLFQGPMVSKGEELFTGVVPILVELDGDVNGHK<br>FSVSGEGEGDATYGKLTLKFICTTGKLPVPWPTLVTTLTYGVQCFSR<br>YPDHMKQHDFFKSAMPEGYVQERTIFFKDDGNYKTRAEVKFEGD<br>TLVNRIELKGIDFKEDGNILGHKLEYNYNSHNVYIMADKQKNGIKV<br>NFKIRHNIEDGSVQLADHYQQNTPIGDGPVLLPDNHYLSTQSALSK<br>DPNEKRDHMVLLEFVTAAGITLGMDELYKGSG**KEAYES**

Supplementary Table 4. Continued.

Protein name	Amino acid sequence
EGFP-Y4A	MHHHHHHHHHLEVLFQGPMVSKGEELFTGVVPILVELDGDVNGHK<br>FSVSGEGEGDATYGKLTLKFICTTGKLPVPWPTLVTTLTYGVQCFSR<br>YPDHMKQHDFFKSAMPEGYVQERTIFFKDDGNYKTRAEVKFEGD<br>TLVNRIELKGIDFKEDGNILGHKLEYNYNSHNVYIMADKQKNGIKV<br>NFKIRHNIEDGSVQLADHYQQNTPIGDGPVLLPDNHYLSTQSALSK<br>DPNEKRDHMVLLEFVTAAGITLGMDELYKGSG**KEGAES**
EGFP-E5A	MHHHHHHHHHLEVLFQGPMVSKGEELFTGVVPILVELDGDVNGHK<br>FSVSGEGEGDATYGKLTLKFICTTGKLPVPWPTLVTTLTYGVQCFSR<br>YPDHMKQHDFFKSAMPEGYVQERTIFFKDDGNYKTRAEVKFEGD<br>TLVNRIELKGIDFKEDGNILGHKLEYNYNSHNVYIMADKQKNGIKV<br>NFKIRHNIEDGSVQLADHYQQNTPIGDGPVLLPDNHYLSTQSALSK<br>DPNEKRDHMVLLEFVTAAGITLGMDELYKGSG**KEGYAS**
EGFP-P0 site Lys	MHHHHHHHHHLEVLFQGPMVSKGEELFTGVVPILVELDGDVNGHK<br>FSVSGEGEGDATYGKLTLKFICTTGKLPVPWPTLVTTLTYGVQCFSR<br>YPDHMKQHDFFKSAMPEGYVQERTIFFKDDGNYKTRAEVKFEGD<br>TLVNRIELKGIDFKEDGNILGHKLEYNYNSHNVYIMADKQKNGIKV<br>NFKIRHNIEDGSVQLADHYQQNTPIGDGPVLLPDNHYLSTQSALSK<br>DPNEKRDHMVLLEFVTAAGITLGMDELYKGSG**KREGYES**
EGFP-P2 site Lys	MHHHHHHHHHLEVLFQGPMVSKGEELFTGVVPILVELDGDVNGHK<br>FSVSGEGEGDATYGKLTLKFICTTGKLPVPWPTLVTTLTYGVQCFSR<br>YPDHMKQHDFFKSAMPEGYVQERTIFFKDDGNYKTRAEVKFEGD<br>TLVNRIELKGIDFKEDGNILGHKLEYNYNSHNVYIMADKQKNGIKV<br>NFKIRHNIEDGSVQLADHYQQNTPIGDGPVLLPDNHYLSTQSALSK<br>DPNEKRDHMVLLEFVTAAGITLGMDELYKGSG**RKGYES**
EGFP-KXGYES	MHHHHHHHHHLEVLFQGPMVSKGEELFTGVVPILVELDGDVNGHK<br>FSVSGEGEGDATYGKLTLKFICTTGKLPVPWPTLVTTLTYGVQCFSR<br>YPDHMKQHDFFKSAMPEGYVQERTIFFKDDGNYKTRAEVKFEGD<br>TLVNRIELKGIDFKEDGNILGHKLEYNYNSHNVYIMADKQKNGIKV<br>NFKIRHNIEDGSVQLADHYQQNTPIGDGPVLLPDNHYLSTQSALSK<br>DPNEKRDHMVLLEFVTAAGITLGMDELYKGSG**KXGYES**<br>(X=L/F/S/R/Q/G)
EGFP-KEGYEX	MHHHHHHHHHLEVLFQGPMVSKGEELFTGVVPILVELDGDVNGHK<br>FSVSGEGEGDATYGKLTLKFICTTGKLPVPWPTLVTTLTYGVQCFSR<br>YPDHMKQHDFFKSAMPEGYVQERTIFFKDDGNYKTRAEVKFEGD<br>TLVNRIELKGIDFKEDGNILGHKLEYNYNSHNVYIMADKQKNGIKV<br>NFKIRHNIEDGSVQLADHYQQNTPIGDGPVLLPDNHYLSTQSALSK<br>DPNEKRDHMVLLEFVTAAGITLGMDELYKGSG**KEGYEX** (X=<br>L/F/R/E/Q/G)
EGFP-N	MHHHHHHHHHLEVLFQGP**KEGYEE**GMVSKGEELFTGVVPILVELD<br>GDVNGHKFSVSGEGEGDATYGKLTLKFICTTGKLPVPWPTLVTTLT<br>YGVQCFSRYPDHMKQHDFFKSAMPEGYVQERTIFFKDDGNYKTR<br>AEVKFEGDTLVNRIELKGIDFKEDGNILGHKLEYNYNSHNVYIMAD<br>KQKNGIKVNFKIRHNIEDGSVQLADHYQQNTPIGDGPVLLPDNHY<br>LSTQSALSKDPNEKRDHMVLLEFVTAAGITLGMDELYK

Supplementary Table 4. Continued.

Protein name	Amino acid sequence
EGFP-I	MHHHHHHHHHLEVLFQGPMVSKGEELFTGVVPILVELDGDVNGHK<br>FSVSGEGEGDATYGKLTLKFICTTGKLPVPWPTLVTTLTYGVQCFSR<br>YPDHMKQHDFFKSAMPEGYVQERTIFFKDDGNYKTRAEVKFEGD<br>TLVNRIELKGIDFKEDGNILGHKLEYNYNSHNVYIMADKQKNGIKV<br>NFKIRHNIEDGSGSG**KEGYEE**GSVQLADHYQQNTPIGDGPVLLPD<br>NHYLSTQSALSKDPNEKRDHMVLLEFVTAAGITLGMDELYK
EGFP-C	MHHHHHHHHHLEVLFQGPMVSKGEELFTGVVPILVELDGDVNGHK<br>FSVSGEGEGDATYGKLTLKFICTTGKLPVPWPTLVTTLTYGVQCFSR<br>YPDHMKQHDFFKSAMPEGYVQERTIFFKDDGNYKTRAEVKFEGD<br>TLVNRIELKGIDFKEDGNILGHKLEYNYNSHNVYIMADKQKNGIKV<br>NFKIRHNIEDGSVQLADHYQQNTPIGDGPVLLPDNHYLSTQSALSK<br>DPNEKRDHMVLLEFVTAAGITLGMDELYKGSG**KEGYEE**
LACE-tagged EGFP	MHHHHHHHHHLEVLFQGPMVSKGEELFTGVVPILVELDGDVNGHK<br>FSVSGEGEGDATYGKLTLKFICTTGKLPVPWPTLVTTLTYGVQCFSR<br>YPDHMKQHDFFKSAMPEGYVQERTIFFKDDGNYKTRAEVKFEGD<br>TLVNRIELKGIDFKEDGNILGHKLEYNYNSHNVYIMADKQKNGIKV<br>NFKIRHNIEDGSVQLADHYQQNTPIGDGPVLLPDNHYLSTQSALSK<br>DPNEKRDHMVLLEFVTAAGITLGMDELYKGSGPRKV**IKME**SEE
α-Synuclein （one SUE1 tag at K43）	MDVFMKGLSKAKEGVVAAAEKTKQGVAEAAGKTKEGVLYVGS**K**<br>**EGYEE**VHGVATVAEKTKEQVTNVGGAVVTGVTAVAQKTVEGAGSI<br>AAATGFIKKEQLGKNEEGAPQEGILEDMPVDPDNEAYEMPSEEGY<br>QDYEPEAC
α-Synuclein （Two SUE1 tags at K43/K96）	MDVFMKGLSKAKEGVVAAAEKTKQGVAEAAGKTKEGVLYVGS**K**<br>**EGYEE**VHGVATVAEKTKEQVTNVGGAVVTGVTAVAQKTVEGAGSI<br>AAATGFI**KEGYEE**KNEEGAPQEGILEDMPVDPDNEAYEMPSEEGY<br>QDYEPEAC
α-Synuclein （one SUE1 tag at K43, one LACE tag at K96）	MDVFMKGLSKAKEGVVAAAEKTKQGVAEAAGKTKEGVLYVGS**K**<br>**EGYEE**VHGVATVAEKTKEQVTNVGGAVVTGVTAVAQKTVEGAGSI<br>AAATGF**IKKE**QLGRNEEGAPQEGILEDMPVDPDNEAYEMPSEEGY<br>QDYEPEAC
p53 (SUE1 tag at K24)	GPMEEPQSDPSVEPPLSQETFSDLW**KEGYEE**NVLSPLPSQAMDDL<br>MLSPDDIEQWFTEDPGPDEAPRMPEAAPPVAPAPAAPTPAAPAPAPS<br>WPLSSSVPSQKTYQGSYGFRLGFLHSGTAKSVTCTYSPALNKMFCQ<br>LAKTCPVQLWVDSTPPPGTRVRAMAIYKQSQHMTEVVRRCPHHER<br>CSDSDGLAPPQHLIRVEGNLRVEYLDDRNTFRHSVVVPYEPPEVGS<br>DCTTIHYNYMCNSSCMGGMNRRPILTIITLEDSSGNLLGRNSFEVRV<br>CACPGRDRRTEEENLRKKGEPHHELPPGSTKRALPNNTSSSPQPKK<br>KPLDGEYFTLQIRGRERFEMFRELNEALELKDAQAGKEPGGSRAH<br>SSHLKSKKGQSTSRHKKLMFKTEGPDSD
p53 (SUE1 tag at K384)	GPMEEPQSDPSVEPPLSQETFSDLWKLLPENNVLSPLPSQAMDDLM<br>LSPDDIEQWFTEDPGPDEAPRMPEAAPPVAPAPAAPTPAAPAPAPSW<br>PLSSSVPSQKTYQGSYGFRLGFLHSGTAKSVTCTYSPALNKMFCQL<br>AKTCPVQLWVDSTPPPGTRVRAMAIYKQSQHMTEVVRRCPHHER<br>CSDSDGLAPPQHLIRVEGNLRVEYLDDRNTFRHSVVVPYEPPEVGS<br>DCTTIHYNYMCNSSCMGGMNRRPILTIITLEDSSGNLLGRNSFEVRV<br>CACPGRDRRTEEENLRKKGEPHHELPPGSTKRALPNNTSSSPQPKK<br>KPLDGEYFTLQIRGRERFEMFRELNEALELKDAQAGKEPGGSRAH<br>SSHLKSKKGQSTSRHKKLMF**KEGYEE**SD

Supplementary Table 4. Continued.

Protein name	Amino acid sequence
NCB1 (one SUE1 tag at K64)	MALRVTRNSKINAENKAKINMAGAKRVPTAPAATSKPGLRPRTALG DIGNKVSEQLQAKMPMK**KEGYEE**ATGKVIDKKLPKPLEKVPMCH HHHHH
Ubiquitin	MQIFVKTLTGKTITLEVEPSDTIENVKAKIQDKEGIPPDQQRLIFAGK QLEDGRTLSDYNIQKESTLHLVLRLRGG
NEDD8	MLIKVKTLTGKEIEIDIEPTDKVERIKERVEEKEGIPPQQQRLIYSGK QMNDEKTAADYKILGGSVLHLVLALRGG
Ufd4	GPMSENNSHNLDEHESHSENSDYMMDTQVEDDYDEDGHVQG EYSYYPDEDEDEHMLSSVGSFEADDGEDDDNDYHHEDDSGLL YGYHRTQNGSDEDRNEEEDGLERSHDNNEFGSNPLHLPDILETF AQRLEQRRQTSEGLGQHPVGRTLPEILSMIGGRMERSAESSARN ERISKLIENTGNASEDPYIAMESLKELSENILMMNQMVVDRIIPM ETLIGNIAAILSDKILREELELQMQACRCMYNLFEVCPESISIAVD EHVIPILQGKLVEISYIDLAEQVLETVEYISRVHGRDILKTGQLSI YVQFFDFLTIHAQRKAIAIVSNACSSIRTDDFKTIVEVLPTLKPIFS NATDQPILTRLVNAMYGICGALHGVDKFETLFSLDLIERIVQLVS IQDTPLENKLKCLDILTVLAMSSDVLSRELREKTDIVDMATRSF QHYSKSPNAGLHETLIYVPNSLLISISRFIVVLFPPEDERILSADK YTGNSDRGVISNQEKFDSLVQCLIPILVEIYTNAADFDVRRYVLI ALLRVVSCINNSTAKAINDQLIKLIGSILAQKETASNANGTYSSE AGTLLVGGLSLLDLICKKFSELFFPSIKREGIFDLVKDLSVDFNNI DLKEDGNENISLSDEEGDLHSSIEECDEGDEEYDYEFTDMEIPDS VKPKKISIHIFRTLSLAYIKNKGVNLVNRVLSQMNVEQEAITEEL HQIEGVVSILENPSTPDKTEEDWKGIWSVLKKCIFHEDFDVSGF EFTSTGLASSITKRITSSTVSHFILAKSFLEVFEDCIDRFLEILQSA LTRLENFSIVDCGLHDGGGVSSLAKEIKIKLVYDGDASKDNIGT DLSSTIVSVHCIASFTSLNEFLRHRMVRMRFLNSLIPNLTSSSTEA DREEEENCLDHMRKKNFDFFYDNEKVDMESTVFGVIFNTFVRR NRDLKTLWDDTHTIKFCKSLEGNNRESEAAEEANEGKKLRDFY KKREFAQVDTGSSADILTLLDFLHSCGVKSDSFINSKLSAKLAR QLDEPLVVASGALPDWSLFLTRRFPFLFPFDTRMLFLQCTSFGY GRLIQLWKNKSKGSKDLRNDEALQQLGRITRRKLRISRKTIFAT GLKILSKYGSSPDVLEIEYQEEAGTGLGPTLEFYSVVSKYFARKS LNMWRCNSYSYRSEMDVDTTDDYITTLLFPEPLNPFSNNEKVIE LFGYLGTFVARSLLDNRILDFRFSKVFFELLHRMSTPNVTTVPSD VETCLLMIELVDPLLAKSLKYIVANKDDNMTLESLSLTFTVPGN DDIELIPGGCNKSLNSSNVEEYIHGVIDQILGKGIEKQLKAFIEGF SKVFSYERMLILFPDELVDIFGRVEEDWSMATLYTNLNAEHGYT MDSSIIHDFISIISAFGKHERRLFLQFLTGSPKLPIGGFKSLNPKFT VVLKHAEDGLTADEYLPSVMTCANYLKLPKYTSKDIMRSRLCQ AIEEGAGAFLLS

Supplementary Table 4. Continued.

Protein name	Amino acid sequence
Ufd2	GPMTAIEDILQITTDPSDTRGYSLLKSEEVPQGSTLGVDFIDTLLL<br>YQLTENEKLDKPFEYLNDCFRRNQQQKRITKNKPNAESLHSTF<br>QEIDRLVIGYGVVALQIENFCMNGAFINYITGIVSNVNSYTDFLS<br>QIIQRAILEGTALDLLNAVFPTLLEYCNKHVSHFDLNESVIYNNV<br>LTIFELFVTFKPIAEIFTKIDGFFADYSCKPQDFERKTILGPILSLSP<br>IEAAVAIRNYGDNLLRSKQQTAMIHESLQAEHKVVIDRLFFIVDK<br>LVRGSLNSRTDMISYFAHIANKNHLRRADHPPFKELSSNGFMSN<br>ITLLLVRFSQPFLDISYKKIDKIDANYFNNPSLFIDLSGETRLNSD<br>FKEADAFYDKNRKTADSKPNFISDCFFLTLTYLHYGLGGTLSFE<br>EKMGSEIKALKEEIEKVKKIAANHDVFARFITAQLSKMEKALKT<br>TESLRFALQGFFAHRSLQLEVFDFICGASTFLIRVVDPEHEFPFKQ<br>IKLPLIPDQIGVENVDNADFLRAHAPVPFKYYPEFVVEGPVNYS<br>LYISKYQTSPIFRNPRLGSFVEFTTMVLRCPELVSNPHLKGKLVQ<br>LLSVGAMPLTDNSPGFMMDIFEHDELVNKNLLYALLDFYVIVEK<br>TGSSSQFYDKFNSRYSISIILEELYYKIPSYKNQLIWQSQNNADFF<br>VRFVARMLNDLTFLLDEGLSNLAEVHNIQNELDNRARGAPPTR<br>EEEDKELQTRLASASRQAKSSCGLADKSMKLFEIYSKDIPAAFV<br>TPEIVYRLASMLNYNLESLVGPKCGELKVKDPQSYSFNPKDLLK<br>ALTTVYINLSEQSEFISAVAKDERSFNRNLFVRAVDILGRKTGLA<br>SPEFIEKLLNFANKAEEQRKADEEEDLEYGDVPDEFLDPLMYTI<br>MKDPVILPASKMNIDRSTIKAHLLSDSTDPFNRMPLKLEDVTPN<br>EELRQKILCFKKQKKEEAKHKASE

Supplementary Table 5. HDX-MS summary table.

Data Set	UBE2E1	UBE2E1+Peptide
HDX reaction details	4 M Gn-HCl, 200 mM citric acid, 500 mM TCEP, pD_{read} = 2.250, 25 °C	4 M Gn-HCl, 200 mM citric acid, 500 mM TCEP, pD_{read} = 2.250, 25 °C
HDX time course	0.5, 1.5, 5, 1440 min	0.5, 1.5, 5, 1440 min
HDX control samples	Maximally-labeled control (UBE2E1)	Maximally-labeled control (UBE2E1)
Back-exchange (mean / IQR)	55.42%/16.3%	
# of Peptides	96	96
Sequence coverage	100%	100%
Average peptide length / Redundancy	14.6/8.2	14.6/8.2
Replicates (biological or technical)	1	1
Repeatability	-	-
Significant differences in HDX (delta HDX > X D)	-	-

Supplementary Methods

1. HPLC purification

All reversed-phase HPLC (RP-HPLC) were performed on Shimadzu Prominence LC-20AT system from Shimadzu company at room temperature. The reactions were monitored and analyzed at both 214 nm and 254 nm wavelengths. For protein analysis, we used Welch Ultimate XB-C18 (4.6 × 250 mm, 5 μm particle size), Welch Ultimate XB-C4 (4.6 mm × 250 mm, 5 μm particle size) and Grace Vydac C4 (4.6 mm × 250 mm, 5 μm particle size) as columns. The flow rate of mobile phase was 1.0 mL/min. The volume of the sample loop was 100 μL or 1 mL. Buffer A was acetonitrile containing 0.1% trifluoroacetic acid (TFA) while buffer B was deionized distilled water (ddH_2O) containing 0.1% TFA. For protein semi-preparative purification, we used Grace Vydac C8 (10 mm× 250 mm, 5 μm particle size) and Welch Ultimate XB-C18 (21.2 mm × 150 mm, 5 μm particle size) as columns. The volume of the sample loop was 5mL.The flow rate of mobile phase varied from 5.0 mL/min to 10.0 mL/min. Buffer A was acetonitrile containing 0.1% TFA while buffer B was deionized distilled water containing 0.1% TFA.

2. Peptide synthesis and ligation methods

List of the protected amino acids blocks used in peptide synthesis

Fmoc-Ala-OH, Fmoc-Arg(Pbf)-OH, Fmoc-Asn(Trt)-OH, Fmoc-Asp(OtBu)-OH, Fmoc-Asp(OMpe)-OH, Fmoc-Cys(Trt)-OH, Fmoc-Glu(OtBu)-OH, Fmoc-Gly-OH, Fmoc-His(Trt)-OH, Fmoc-Ile-OH, Fmoc-Leu-OH, Fmoc-Lys(Boc)-OH, Fmoc-Met-OH, Fmoc-Phe-OH, Fmoc-Pro-OH, Fmoc-Ser(tBu)-OH, Fmoc-Thr(tBu)-OH,Fmoc-Trp(Boc)-OH, Fmoc-Tyr(tBu)-OH, Fmoc-Val-OH, Fmoc-auxiliary-Gly-OH.

General procedure of microwave-assisted Solid-Phase Peptide Synthesis

All peptides in this paper were synthesized via Liberty Blue Automated microwave peptide synthesizer (CEM Corporation, U.S.A.), following the standard 9-fluorenyl-methoxycarbonyl (Fmoc)-based solid phase peptide synthesis (SPPS) protocol. All

reaction amino acid blocks, coupling reagents and deprotection reagent were dissolved in DMF. The concentration of all reaction amino acid blocks was 0.2 M. The coupling reagents were DIC and Oxyma while the concentration of DIC and Oxyma stocks were 1.4 M and 0.7 M, respectively. The deprotection reagent was 10% piperidine dissolved in DMF with a final concentration of 0.1 M Oxyma in order to reduce racemization during the reaction. The dosage relationship of each component is resin: amino acid: DIC: Oxyma=1: 4: 8: 4.

The process of each coupling reaction cycle was:
1) Resin swelling: 10 mL of DMF was added to the reaction tube, swelled the resin for 10 minutes, and then drained the DMF.
2) Deprotection: 7 mL of deprotection reagent was pipetted into the reaction tube, the reaction tube was heated to 90°C by microwave for 1.5 minutes, wash three times by 7ml DMF and then drained the solvent.
3) Coupling: DIC (1.5 mL), Oxyma (1.5 mL) and amino acid stocks (5 mL) were pre-activated first. Then the mixture was added to the reaction tube, heated to 90 °C by microwave for 3 minutes. Then the resin was washed by DMF three times followed by draining of the solvent. Repeating steps 2-3 to achieve the elongation of amino acids on the resin.
4) Final deprotection: Once all the amino acid coupling was completed, repeated step one to achieve the removal of N-terminal Fmoc protecting group. (Note: Amino acids such as Arginine, Valine, Proline and any amino acid after Proline, or specific sequences would be coupled twice during the coupling process; The reaction temperature of Cys, His in the coupling step was 50°C and the reaction time was extended to 10 min.)

Once the peptide was completed synthesized, the resin was transferred to a synthetic tube with sand core, washed by DMF and DCM. After draining the solvent, 20mL of cleavage cocktail (ratio of H_2O: TIPS: anisole sulfide: EDT: TFA=5: 5: 5: 3: 82) was added to the synthetic tube. Then shaking the synthetic tube for 2 hours at room

temperature. After cleavage, the crude peptides were separated out by precipitation with pre-chilled ether.

Native chemical ligation (NCL)

The hydrazide fragment (1.0 eq.) was first placed in the reaction tube, dissolved in the reaction buffer (6 M guanidine hydrochloride, 100 mM NaH_2PO_4, pH 2.3). The reaction solution was then pre-cooled in Dewar flask at -15°C ice salt bath for 5 minutes followed by the addition of $NaNO_2$ (10.0 eq). After the hydrazide fragment was oxidized for 15 to 20 minutes, exceed MPAA (60.0 eq) was added to the reaction. Once MPAA was introduced, the reaction system could be taken out from the ice salt bath to the room temperature and the pH of the reaction mixture was slowly adjusted to 5.1 using 2 M NaOH aqueous solution. Stirring at room temperature for 5 minutes, the second fragment with N-terminal cysteine dissolved in the buffer (6 M guanidine hydrochloride, 100 mM Na_2HPO_4, pH 6.8) was then added to the reaction mixture. Finally, the pH of the reaction system was slowly adjusted to 6.4 using 2 M NaOH aqueous solution. The mixture reacted at room temperature while the progress of the reaction was monitored by HPLC and mass spectrometry.

The desulfurization of proteins

The peptide fragment (1.0 eq.) containing cysteine was first dissolved in desulfurization buffer (6 M guanidine hydrochloride, 100 mM Na_2HPO_4, 300 mM TCEP, pH 7.0) with the final concentration of 0.5g mL^{-1}. Then tBuSH (1000 eq) was added to the reaction mixture followed by the initiator VA-044 (17mg/mL). Finally, the reaction mixture was stirred at 37 °C for 5 hours and monitored by analytical HPLC until the peptide was desulfurized completely. Once the reaction was finished, an equal volume of buffer (6 M guanidine hydrochloride, 100 mM NaH_2PO_4, pH 2.3) was added to the reaction mixture followed by the semi-preparative purification using HPLC.

3. The preparation of $Ub_{1\text{-}75}$-$NHNH_2$

First, Ubiquitin carrying the G76C mutation obtained by recombinant expression was concentrated to 15mg mL^{-1}. Guanidine hydrochloride was then added to the solution as the final concentration was 6 M. Next, MesNa, TCEP and hydrazine hydrochloride were added to the solution as the final concentration was 10mg mL^{-1}, 5mg mL^{-1} and 5 mg mL^{-1}, respectively. The pH of the reaction mixture was then adjusted to 6.5 followed by the seal of the reaction vessel. The reaction vessel was then shaken at 50°C for 48 hours and the reaction was monitored by analytical HPLC. Once the starting material was completely converted to the product, the reaction was suspended and the products were separated by semi-preparative HPLC.

4. Mass spectrometry analysis of ubiquitin (or NEDD8) modification sites

The gel bands of interest were separated from the gel and then subjected to an in-gel digestion at 37 °C overnight. The peptides were then extracted twice with buffer (0.1% trifluoroacetic acid and 50% aqueous acetonitrile) and dried using a SpeedVac (Thermo Scientific). 0.1% aqueous trifluoroacetic acid was used to redissolve the peptides and analyzed by Thermo orbitrap fusion. For LC-MS/MS analyses, a gradient elution (0.30 µl min-1) was performed for 60 min to separate the peptides using an EASY-nLC 1000 system connected directly to an Orbitrap Fusion Tribrid mass spectrometer (Thermo Fisher). The Orbitrap Fusion mass spectrometer was operated in data-dependent acquisition mode using Xcalibur 3.0 software. A single full scan mass spectrum (350-1550 m/z, 120,000 resolution) was acquired, followed by top-speed MS/MS scans in the Orbitrap. An in-house Proteome Discoverer (Version PD1.4, Thermo-Fisher) was used to search the MS/MS spectra from each LC-MS/MS run based on the target protein sequence. The search settings were as follows: mass tolerance of the precursor ions was set to 20 ppm for all MS; mass tolerance of the fragment ions was set to 0.02 Da for all acquired MS2 spectra; Using the fixed value PSM validator to calculate the peptide false discovery rate (FDR) and a peptide spectrum match (PSM) is considered correct if the q value is less than 1%.

5. Hydrogen/deuterium exchange mass spectrometry (HDX-MS)

UBE2E1 at a final concentration of 100 μM was incubated with 5-fold equivalents hexapeptide（KEGYES）at 4°C. This mixture was then diluted 10 times with D_2O solution (50 mM HEPES, pD 7.5 150 mM NaCl, 99% D_2O) to initiate the deuterium exchange. At time points (0.5, 1.5, 5 min) , hydrogen/deuterium exchange reaction was quenched by the addition of buffer (4 M Gn-HCl, 200 mM citric acid, 500 mM TCEP, pH 1.8) and then subjected to digestion by pepsin for 3 min. Next, the peptides were separated and analyzed using a Thermo Scientific Q Exactive connected to a Thermo-Dionex Ultimate 3000 HPLC system. HDX-MS data were processed and analyzed using HDExaminer (v.PD1.4, ThermoFisher Scientific).
