# Supplementary material for: Structure-guided engineering enables E3 ligase-free and versatile protein ubiquitination via UBE2E1
Source: Nat Commun. 2024 Feb 10;15:1266. doi: 10.1038/s41467-024-45635-y (PMC10858943; doi:10.1038/s41467-024-45635-y)
Supplement: Supplementary file 3 — Reporting Summary [file 41467_2024_45635_MOESM3_ESM.pdf]

nature research

Corresponding author(s): Lei Liu

Last updated by author(s): Dec 28, 2023

Reporting Summary

Nature Research wishes to improve the reproducibility of the work that we publish. This form provides structure for consistency and transparency in reporting. For further information on Nature Research policies, see Authors & Referees and the Editorial Policy Checklist.

Statistics

For all statistical analyses, confirm that the following items are present in the figure legend, table legend, main text, or Methods section.

n/a	Confirmed	
☐	☒	The exact sample size (*n*) for each experimental group/condition, given as a discrete number and unit of measurement
☐	☒	A statement on whether measurements were taken from distinct samples or whether the same sample was measured repeatedly
☒	☐	The statistical test(s) used AND whether they are one- or two-sided<br>*Only common tests should be described solely by name; describe more complex techniques in the Methods section.*
☒	☐	A description of all covariates tested
☒	☐	A description of any assumptions or corrections, such as tests of normality and adjustment for multiple comparisons
☒	☐	A full description of the statistical parameters including central tendency (e.g. means) or other basic estimates (e.g. regression coefficient) AND variation (e.g. standard deviation) or associated estimates of uncertainty (e.g. confidence intervals)
☒	☐	For null hypothesis testing, the test statistic (e.g. *F*, *t*, *r*) with confidence intervals, effect sizes, degrees of freedom and *P* value noted<br>*Give P values as exact values whenever suitable.*
☒	☐	For Bayesian analysis, information on the choice of priors and Markov chain Monte Carlo settings
☒	☐	For hierarchical and complex designs, identification of the appropriate level for tests and full reporting of outcomes
☒	☐	Estimates of effect sizes (e.g. Cohen's *d*, Pearson's *r*), indicating how they were calculated

Our web collection on statistics for biologists contains articles on many of the points above.

Software and code

Policy information about availability of computer code

Data collection	X-ray: Finback; Gel Imaging: Image Lab Touch Software-2.4.0.03; Mass spectrometry: Thermo Scientific Orbitrap Fusion and Thermo Scientific Q Exactive
Data analysis	X-ray: Aimless-0.7.7, PHENIX-1.19.2; Model Building: PHENIX-1.19.2, COOT-0.8.2; Molecular Visualization: ChimeraX-1.2.5, PyMol-2.3.0; Gel Bands Quantification: Image Lab-6.0.1; Curve Fitting: OriginPro 9, GraphPad Prism-9.0; MS: HDExaminer-v.PD1.4 and a in-house Proteome Discoverer (Version PD1.4, Thermo-Fisher)

For manuscripts utilizing custom algorithms or software that are central to the research but not yet described in published literature, software must be made available to editors/reviewers. We strongly encourage code deposition in a community repository (e.g. GitHub). See the Nature Research guidelines for submitting code & software for further information.

Data

Policy information about availability of data

All manuscripts must include a data availability statement. This statement should provide the following information, where applicable:

- Accession codes, unique identifiers, or web links for publicly available datasets
- A list of figures that have associated raw data
- A description of any restrictions on data availability

The coordinates and structure factor files for UBE2E1-SETDB1 derived peptide complex have been deposited in the Protein Data Bank (PDB) under accession number 8IYA (https://doi.org/10.2210/pdb8IYA/pdb). The HDX-MS and MS/MS data have been deposited to the ProteomeXchange Consortium via the PRIDE partner repository with the dataset identifier PXD048115 and PXD048116.The atomic model of UBE2E1 is available under PDB accession code 3BZH [https://doi.org/10.2210/pdb3bzh/pdb] and 5LBN [https://doi.org/10.2210/pdb5LBN/pdb]. The atomic model of α-Synuclein is available under PDB accession code 1XQ8 [https://doi.org/10.2210/pdb1XQ8/pdb]. The predicted atomic model of p53 is available in the AlphaFold Protein Structure Database under accession code AF-P04637-F1 [https://alphafold.ebi.ac.uk/entry/P04637]. Source data are provided with this paper.

Field-specific reporting

Please select the one below that is the best fit for your research. If you are not sure, read the appropriate sections before making your selection.

☒ Life sciences ☐ Behavioural & social sciences ☐ Ecological, evolutionary & environmental sciences

For a reference copy of the document with all sections, see nature.com/documents/nr-reporting-summary-flat.pdf

Life sciences study design

All studies must disclose on these points even when the disclosure is negative.

Sample size	The work performed here did not involve statistical analyses that would be impacted by sample size.
Data exclusions	No data were excluded from the analysis.
Replication	The ubiquitination assay to investigate the effect of mutations in the second and sixth positions (on the hexapeptide) , and the kinetic data of the ubiquitination reaction were performed with three independent biological replicates. The proteasomal degradation assay were performed with three independent biological replicates and the corresponding control experiment was repeated twice. All attempts at replication were successful.
Randomization	Randomization was not relevant to this study because no grouped samples were involved.
Blinding	Blinding was not relevant to this study because no grouped samples were involved.

Reporting for specific materials, systems and methods

We require information from authors about some types of materials, experimental systems and methods used in many studies. Here, indicate whether each material, system or method listed is relevant to your study. If you are not sure if a list item applies to your research, read the appropriate section before selecting a response.

Materials & experimental systems

n/a	Involved in the study	
☒	☐	Antibodies
☒	☐	Eukaryotic cell lines
☒	☐	Palaeontology
☒	☐	Animals and other organisms
☒	☐	Human research participants
☒	☐	Clinical data

Methods

n/a	Involved in the study	
☒	☐	ChIP-seq
☒	☐	Flow cytometry
☒	☐	MRI-based neuroimaging
